# The impact of educational live action role-playing games on social–emotional competence: a mixed-method study with Chinese college students

**DOI:** 10.3389/fpsyg.2025.1538761

**Published:** 2025-06-09

**Authors:** Jiayi Zhang, Jing Xu, Muru Li

**Affiliations:** ^1^Department of Law and Political Science, North China Electric Power University, Baoding, China; ^2^Institute of Education, Sichuan University, Chengdu, China

**Keywords:** educational live action role-playing games, social–emotional competence, collaborative learning, mixed-method study, college students

## Abstract

**Background:**

Social–emotional competence is critical for university students’ personal development and social adaptation, and exploring effective educational interventions in this regard is an important research area. Live action role-playing games (LARPs) have shown potential in promoting social skills and emotional regulation, but their specific impacts on Chinese university students remain largely unexplored.

**Objective:**

This mixed-method research was designed to explore the influence of real-time educational role-playing games (LARPs) on the social–emotional competence of Chinese university students.

**Methods:**

A sample of 84 participants was recruited, with 42 assigned to the control group and 42 to the experimental group. Over an 8-week period, the experimental group engaged in the educational live action role-playing games project for 2–5 h per week. To comprehensively evaluate the social–emotional competence of the participants, the Social–Emotional Competence Questionnaire was employed for quantitative assessment. Additionally, in-depth one-on-one interviews were employed within a qualitative research design to conduct thematic analysis. Based on quantitative and qualitative research, an explanatory sequential design was adopted to comprehensively identify the research findings.

**Results and Discussion:**

The results demonstrated that, compared with the control group, the experimental group exhibited a significant increase in social–emotional competence. In the dimensions of Engaging with others, Collaboration, and Open-mindedness, the improvements were remarkable. Further in-depth analysis indicated significant progress in specific sub-dimensions within each competency. In Engaging with others, Assertiveness and Sociability were enhanced; in Collaboration, Empathy and Co-operation were improved; and in Open-mindedness, Tolerance and Curiosity showed growth. Both the qualitative and quantitative data consistently suggest that educational live action role-playing games can arouse the interest of a wide range of students and effectively enhance their social–emotional competence. Drawing from the results, this research offered pedagogical and analytical insights to enhance the integration of educational live action role-playing games into co-curricular programs at the higher education context: providing high-quality scenario scripts with pedagogical significance, creating immersive contextually authentic environments, and implementing professional development programs for facilitators. This research also provided a new perspective on integrating game-centered elements and cooperative mechanisms into official curricula.

## 1 Introduction

One of the most important challenges facing education is how to cultivate greater talents for society. The Global Sustainable Development Goals (SDG) which were advocated by the United Nations in 2020 are considered a highly influential framework for educational institutions and educators (Guterres, 2020). Among the global sustainable development goals, Goal 4 particularly addresses the progress toward quality education. Target 4.3 mentions ‘providing quality higher education’. Target 4.4 refers to ‘increasing the relevant skills needed for employment and entrepreneurship,’ whereas Target 4.7 emphasizes ‘ensuring that learners acquire the knowledge and skills needed for sustainable development’ (Kioupi and Voulvoulis, 2019; Mogensen and Schnack, 2010). These initiatives prioritize the cultivation of sustainable development skills among college students in higher education (Aver et al., 2021; Delbeke et al., 2019; Trevors and Saier, 2010; Weiland et al., 2021).

Social–emotional competence has been recognized as an essential capability of students which is concerned by the Organization for Economic Co-operation and Development (OECD) in recent years. Its main focus is to determine the specific qualities that students need to develop in order to effectively navigate the challenges and uncertainties of the future (Eva, 2021).

The OECD defines social and emotional competence as the comprehensive capabilities of individuals, conceiving it more as a skill rather than a character trait, emphasizing that it is cultivable and malleable in 2019. In light of the ongoing evolution of social and economic growth, the significance and influence of socio-emotional competence are progressively growing (Belfield et al., 2015; Durlak et al., 2011).

Existing research suggests that impeding the growth of social–emotional competence in adolescents will directly affect on various physical and psychological indicators. These indicators include educational expectations, academic performance, civic participation, physical and mental health, life satisfaction, and subjective well-being (Durlak et al., 2011; Osher et al., 2016; Poulou, 2017). Therefore, exploring pathways to enhance social–emotional competence will assist young individuals in improving their overall capabilities (Taylor et al., 2017), enabling them to effectively interact with and contribute to society, while also helping schools further develop sustainable education and promote social progress.

As for social–emotional competence, previous research on social–emotional competence in college students has been extensive. Mayer’s emotional intelligence model, which defines emotional intelligence as the ability to perceive, use, understand, and manage emotions, has been widely applied in studying college students’ social–emotional development (Mayer, 2000). For instance, studies by Brackett et al. have shown that higher emotional intelligence in college students is associated with better academic performance and social relationships (Brackett et al., 2006). Regarding role-playing games, research has indicated their potential in enhancing various skills. For example, a study by Connolly et al. on digital games found that certain types of role-playing games can improve problem-solving and strategic thinking skills (Connolly et al., 2012). Another study by Schonert-Reichl et al. (2015) implemented a mindfulness-based intervention program, which led to increased empathy and reduced stress levels among college students. These studies provide strong theoretical and empirical support for our research on the impact of educational live-action role-playing games on college students’ social–emotional competence.

In March 2024, researchers recruited 84 student participants of North China Electric Power University through campus posters. This research aims to investigate the role of live action role-playing games in enhancing social–emotional competence among Chinese university students through quantitative research and one-on-one interviews. The research questions addressed are as follows: (1) How can live action role-playing games improve university students’ social–emotional competence? (2) What specific aspects of social–emotional competence can be enhanced by implementing live action role-playing games? Furthermore, we propose measures that schools, teachers, and administrative staff can take to improve the effectiveness of implementing live action role-playing games in order to provide higher-quality education for university students. The research findings will not only contribute to improving the effectiveness of live action role-playing games in enhancing students’ social–emotional competence and educational experience but also provide valuable references for the future implementation of live action role-playing games in higher education institutions.

## 2 Literature review

### 2.1 Research on social–emotional competence

In the face of the complex and ever-changing modern society, the development of individuals’ social–emotional competence has become an important research area in academia. Reviewing past studies, the research on social–emotional competence has primarily focused on adolescent groups and has centered on the following aspects.

Firstly, the research on the effects of students’ social–emotional competence. Relevant scholars have used causal analysis to discuss the impact of social–emotional competence on the enhancement of students’ academic achievement (Liu et al., 2022), the promotion of social development (Taylor et al., 2017; Zhang, 2014; Zhang et al., 2012), the improvement of mental health (Huang et al., 2021), and the inhibition of maladaptive behaviors (He et al., 2019).

Secondly, the research on the influencing factors of students’ social–emotional competence. Currently, the influencing factors of students’ social–emotional competence mainly involve three levels: individual, family, and school. At the individual level, factors such as boarding, migration, and preschool education experiences affect the development of students’ social–emotional competence (Corcoran et al., 2020; Gong and Li, 2018; Yu et al., 2020). Some studies focus on the social–emotional competence of students in rural and western regions (Yang et al., 2019). At the family level, socioeconomic status is the most commonly used variable representing family capital in research, and it has a significant impact on students’ social–emotional competence (Zhang and Zhao, 2022). Some scholars have studied the impact of parental upbringing styles (McWayne et al., 2004), parent–child separation (Mao et al., 2020), and parent–child relationships on children’s social–emotional competence (Ren et al., 2019). At the school level, some researchers believe that class size and classroom atmosphere are important factors affecting students’ social–emotional competence (Li et al., 2021). Additionally, factors such as teachers’ emotions and teaching methods also significantly influence students’ social–emotional competence (Jennings and Greenberg, 2009). Furthermore, some scholars have specifically studied the role of school-family cooperation methods on students’ social–emotional competence (Li and Zhang, 2022).

Thirdly, research on the cultivation of students’ social–emotional competence. Some experimental studies rigorously verify the effectiveness of relevant factors or interventions (primarily at the school level) in enhancing students’ social–emotional competence. For instance, researchers have found that group counseling and peer effects of class leaders can improve students’ social–emotional competence (Yankun, 2014). Yu et al. (2020) studied how combined school-family education for children of migrant workers helps improve their non-cognitive skills. A commonly used method to support the development of students’ social–emotional competence is school-based interventions (Durlak et al., 2011; Dusenbury et al., 2015; Greenberg et al., 2003). Numerous intervention programs abroad aim to promote the social–emotional competence of primary and secondary school students, such as RULER, ZIPPY’s Friends, INSIGHTS, and PATHS (Liaojian and Minxuan, 2023). In China, Mao Yaqing’s research team has implemented the SEL program using a ‘whole-school implementation’ model, which has been carried out in over 500 primary and secondary schools across 11 provinces (Xiaoting, 2021). Given the significant impact of teachers’ instruction on students’ social–emotional competence, some literature has examined the effects of teacher social–emotional competence training programs like ‘CARE’ on teachers and the classes they lead (Mingwei and Yuqing, 2023).

Fourthly, research on measurement tools for students’ social–emotional competence. First, the framework developed by academic and social–emotional learning organizations (CASEL) includes five components: self-awareness, self-management, social awareness, relationship skills, and responsible decision-making (Jones and Doolittle, 2017; Mantz et al., 2018). Second, the “Three Aspects and Six Dimensions” framework established by Mao et al. (2020) during the implementation of SEL projects in China combines Chinese cultural and educational realities. This framework encompasses dimensions related to self, others, and the collective, including self-cognition, self-management, other-cognition, other-management, collective-cognition, and collective-management (Li et al., 2021; Yang et al., 2019). Third, the psychological measurement model represented by the “Big Five Personality” model provides applicable measurement tools through the OECD’s SSES project. The East China Normal University project team localized the questionnaire for use in China, constructing a measurement model that includes five dimensions: task capability, emotional regulation, collaboration ability, openness ability, and communication ability. Due to the challenges of subjectivity and cross-cultural variations in social–emotional competence, individual understanding, as well as social and cultural expectations, can affect the assessment of social–emotional competence. It is necessary to revise and construct measurement tools based on local contexts to enhance cultural adaptability. Some regions also have their own implementation frameworks; for example, the European Union has developed a social–emotional education evaluation system covering three dimensions: personal, social, and learning to learn (Chao and Xianglan, 2023).

There has been some research on social–emotional competence among university students. Yue (2021) qualitatively analyzed the components and realization of college students’ social–emotional adaptability. Ying and Xinxu (2022) qualitatively examined the manifestations of deficiencies and acquisition pathways of social–emotional competence among graduate students. Liaojian et al. (2022) investigated the impact of undergraduate students’ social–emotional competence on their learning engagement. Zhaojun et al. (2023), based on practical considerations, studied the theoretical model and scale development of social–emotional competence for university students, building on existing measurement models.

### 2.2 Research on live action role-playing games

The name “live action role-playing games” originates from “murder mystery,” a social game popular in European and American parties. In these games, multiple players role-play characters from a script, freely interpreting content, advancing the plot, and collaboratively deducing the identity of a perpetrator in a tabletop setting. In 2013, a live action role-playing game called “Death Wears White” was introduced to China, marking the beginning of the live action role-playing games industry in the country (Mei and Ji, 2022). As these games became popular, their themes span various dimensions, including science fiction, ancient fantasy, horror, and more. Coupled with diverse gameplay elements such as logical deduction, audiovisual stimulation, and emotional expression, they have captured the interest of adolescent players. In China, live action role-playing games are an emerging field, with research on this topic only beginning to appear in 2021. Currently, there is a strong growth trend.

Due to the strong immersion and interactivity of live action role-playing games (Bowman and Standiford, 2016; Márquez Segura et al., 2019), some scholars have applied role-playing to psychological therapy or educational purposes (Gershkovich et al., 2017; Wright et al., 2020). Owing to the characteristic of team collaboration inherent in role-playing games, within the domain of psychological treatment, relevant intervention experimental studies have emerged, which lend support to the application of RPGs for the treatment of anxiety, the enhancement of mental well-being, as well as the cultivation of adaptive behaviors (Abbott et al., 2022; Varrette et al., 2023). Internationally, the implementation of educational live action role-playing games began early, with specialized organizations—including consulting firms and after-school programs—emerging to focus on this area (Dagan et al., 2019). Research indicates that traditional learning can be somewhat passive, while role-playing, with its high degree of openness, helps to increase student engagement and initiative (Cole et al., 2020). In the field of scientific curriculum learning, studies by Bowman et al. have also shown that educational live action role-playing games enhance students’ scientific cognitive abilities and improve their overall learning experiences (Bowman and Standiford, 2015). In educational live action role-playing games, students are motivated by both the emotional and narrative elements of the game, as well as the competitive aspects (Gjedde, 2013). Compared to traditional education, these games can enhance students’ self-awareness and intrinsic motivation (Hammer et al., 2018). In terms of social and collaborative skills, research shows that educational live action role-playing games can improve students’ leadership abilities, communication skills, depth of understanding, and teamwork (Guenthner and Moore, 2005). Regarding open-mindedness, research by Pitkänen (2015) indicates that students can enhance empathy [51] and significantly increase tolerance for diversity and empathetic abilities (Howes and Cruz, 2009).

In terms of research methods, early studies were mainly qualitative, focusing on clarifying key concepts (Adams, 2013; Daniau, 2016). Some research teams also pioneered experimental designs, conducting research by comparing the differences in pre-test and pro-test results. Guli et al. (2013) conducted a two-phase controlled intervention experiment on 39 adolescents to study the impact of a creative drama program on adolescents with social difficulties. The results showed that the intervention group had increased positive interactions and improved interpersonal relationships (Guli et al., 2013). Sandstrom et al. (2022) investigated the effect of online role-playing games on alleviating people’s social barriers. They carried out a one-week comparative experiment on 454 individuals, and the results indicated that online role-playing games can reduce the pessimism about social rejection and enhance their ability to communicate (Sandstrom et al., 2022). Abbott et al. (2022) conducted a one-year intervention experiment, using tabletop role-playing games as a medium to provide treatment for 7 adults. Qualitative research showed that the group members improved their anxiety, were able to express their needs confidently, and faced conflicts properly (Abbott et al., 2022). In order to explore the impact of embedding Cognitive Behavioral Therapy (CBT) into tabletop role-playing games (TTRPG) on adults’ social skills, anxiety symptoms, and mental health, Varrette et al. (2023) carried out an experiment lasting 12 weeks (some groups lasted for 24 weeks), with results showing that the subjects had improved symptoms of social anxiety and self-reports indicating that the game process enhanced their social confidence and promoted the practice of social skills. Sancho et al. (2009) conducted a one-semester follow-up experiment on 250 college students using Nucleo (a collaborative 3D fantasy virtual learning scenario). The results showed that this project, which incorporates role-playing elements, is effective in enhancing students’ motivation and cultivating collaboration abilities (Sancho et al., 2009). To evaluate the impact of virtual reality technology on the emotional intelligence of college students, Wang and Pan (2024) conducted a controlled experimental study on 356 college students. The results showed that the experimental group had improved abilities in self-emotion assessment and emotional regulation, confirming the advantages of VR technology in creating immersive experiences and enhancing engagement (Wang and Pan, 2024). Previous studies have explored the impacts of creative drama, online role-playing, tabletop role-playing, and virtual reality technology on social interaction and related psychological aspects, which provides a foundation for exploring on the impact of live action role-playing games on enhancing college students’ social–emotional skills.

Although the role-playing teaching method has emerged as an innovative educational paradigm attracting extensive scholarly attention in global higher education, its localized implementation within Chinese higher education has not been fully explored yet. Current research has primarily focused on establishing the foundational theory of educational drama, examining dimensions such as educational value (Tan, 2023), curriculum attributes (He and Dan, 2025), core characteristics (Sun, 2023), and design strategies (Jiang and Peng, 2025). Scholars have further investigated its theoretical applications across various domains, including disciplinary literacy (Chi and Dong, 2024; He and Xu, 2025), aesthetic education literacy (Xu and Fan, 2024), and mental health at the theoretical level (Wang et al., 2021). In terms of quantitative research, Wang et al. (2021) demonstrated through eight psychodrama intervention experiments that this method can enhance psychological resilience among academically underperforming college students (Wang et al., 2021). Similarly, Liu et al. (2016) verified through six-week interventions that psychodrama techniques can effectively reduce college students’ interpersonal hostility, anxiety, withdrawal tendencies, and distrust. However, it should be noted that the psychodrama methodologies employed in these studies more closely resemble thematic seminars or group counseling sessions than authentic role-playing experiences. Specifically, these interventions exhibit lower levels of role immersion, narrative coherence, and interaction complexity compared to LARP approaches. Methodologically, existing research exhibits two notable limitations: first, a paucity of qualitative investigations into students’ subjective experiences during role-playing activities; second, an insufficient distinction between conventional psychodrama techniques and more sophisticated LARP methodologies. The study of educational LARPs in China is still evolving, necessitating more systematic and in-depth research to refine and advance this pedagogical approach.

In summary, social–emotional skills are crucial for sustainable human development, and educational live action role-playing games offer potential to enhance these skills among college students. This case study analyzes the effects of live action role-playing games on the social–emotional skills of Chinese college students through pre- and post-assessment comparisons and one-on-one interviews, aiming to provide references for the implementation of live action role-playing games in college education.

## 3 Methodology and study design

### 3.1 Mixed-method: explanatory sequential design

In this study, we adopted an explanatory sequential design within the mixed-methods approach, which is a well-recognized and extensively used framework in social science research (Creswell and Clark, 2017; Tashakkori and Teddlie, 2010). This explanatory sequential design is a type of mixed-methods design where quantitative data collection and analysis are conducted first, followed by qualitative data collection and analysis. The purpose is to use the qualitative data to explain and expand upon the quantitative findings. Previous research has effectively utilized this framework. For example, in a study by Johnson et al. (2007), they first collected quantitative data through surveys to identify general trends in students’ academic performance. Then, they conducted qualitative interviews to explore the underlying reasons for those trends, such as students’ study habits and motivation. This approach allowed them to gain a more in-depth understanding of the research topic. Another study (Greene et al., 1989) used the explanatory sequential design to first quantify the impact of a new teaching method on students’ test scores. Subsequently, qualitative focus groups were employed to understand how students experienced the new teaching method, which provided rich context and detailed explanations for the quantitative results.

**Figure 1:**
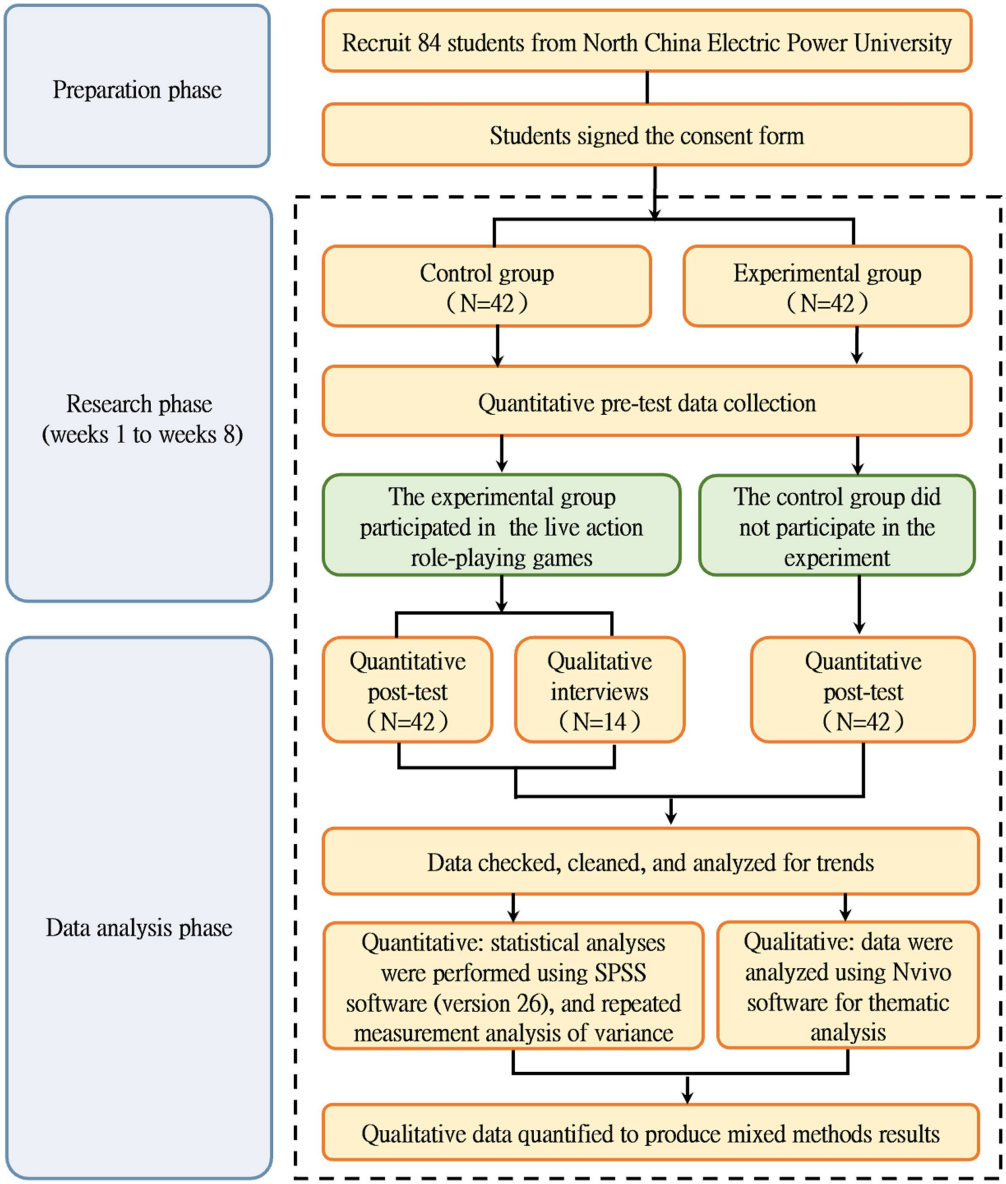
Research flow chart.

In the context of our study, we first gathered quantitative data using the “Social and Emotional Competence Questionnaire.” The numerical results from this quantitative analysis provided us with an overall understanding of the changes in students’ social–emotional competence, revealing trends and significant differences between the experimental and control groups. For instance, we were able to identify which dimensions of social–emotional competence showed significant improvement in the experimental group through statistical analysis. Subsequently, we conducted in-depth qualitative interviews. The qualitative data obtained through these interviews served to explain and expand on the quantitative findings. This sequential approach allowed us to analyze the data from both methods, enhancing the validity and reliability of our research findings. The explanatory sequential design can be defined as a mixed-methods research design in which an initial quantitative phase is followed by a qualitative phase. The quantitative data is used to identify patterns, trends, and relationships, while the qualitative data is then collected and analyzed to explain, elaborate, and contextualize the quantitative results. It aims to integrate the strengths of both quantitative and qualitative research methods, using the objectivity and generalizability of quantitative data and the depth and richness of qualitative data to provide a more comprehensive and in-depth understanding of the research problem. The overview of the study design is shown in Table 1 and the research flow chart is shown in Figure 1.

**Table 1:** Overview of the study design based on a mixed-method approach.

Study	Component	Participants	Data collection	Analysis
Main study	Quantitative	College students (*N* = 84) Experimental group (*N* = 42) Control group (*N* = 42)	Collect and analyze the scores on the social–emotional competence scale for college students in the control group and experimental group before and after the experiment, and examine the indicator data of college students’ social–emotional competence.	Perform statistical analysis using SPSS software (version 26). Refer to 3.2.4 Data Analysis for details
Embedded study	Qualitative	College students (*N* = 28) Experimental group (*N* = 14) Control group (*N* = 14)	Conduct in-depth interviews before and after the experiment according to the pre-designed one-on-one interview outline.	Using NVIVO software conduct thematic induction and analysis.

### 3.2 Quantitative research

#### 3.2.1 Participants

In March 2024, researchers recruited 84 participants through campus posters, with student participants from North China Electric Power University. The researchers were approved by the university to implement this plan. The main investigators of the project were teachers from the Psychological Health Center of North China Electric Power University. Some student research assistants were selected from student organizations to join the project. Student participants were informed of the research plan and signed the informed consent form before the experiment began.

A total of 84 students participated in this quantitative test, including 42 from the experimental group and 42 from the control group. They all attended the tests before and after the end of the experiment (Table 2).

**Table 2:** Demographic information of the experimental and control groups.

Student’s information	Experimental Group (*N* = 42)		Control Group (*N* = 42)	
	*N*	%	*N*	%
Gender	42	100	42	100
Male	20	47.62	19	45.24
Female	22	52.38	23	54.76
Grade	42	100	42	100
Freshman	25	59.52	23	54.76
Sophomore	16	38.10	17	40.48
Junior	0	0	1	2.38
Senior	1	2.38	1	2.38

#### 3.2.2 Data collection

For the quantitative component of this study, the research was conducted utilizing the “Social and Emotional Competence Questionnaire” (Chernyshenko et al., 2018). This questionnaire is derived from the social–emotional competence framework meticulously designed by OECD (Kankaraš and Suarez-Alvarez, 2019). This framework is based on the “Big Five Personality” theory and the ecosystem theory and encompasses five critical dimensions. After 2 years of comprehensive preparation, the OECD conducted the Survey on Social and Emotional Skills (SSES) in 2019, implementing this questionnaire in 10 global cities, including Columbia (United States) and Suzhou (China) (Development, 2021). The extensive geographical coverage and multinational participation underscore the measurement tool’s high recognition and widespread application in social science research. According to the official release of the actual test results in Suzhou, the Cronbach’s *α* coefficients of each dimension of the scale are between 0.73 and 0.85, indicating good reliability. Furthermore, the comparative fit index (CFI = 0.94) and root mean square error of approximation (RMSEA = 0.07) confirmed good validity (Shuzhen, 2023; Zhang et al., 2021). The questionnaire comprises two distinct sections: demographic information and student self-assessment scale, which can be found in Annex 1.

Considering the actual scale of the experiment implementation and based on a review of the existing research literature, this study centers on the potential impact of LARPs on enhancing the socio-emotional competencies of college students. Three primary dimensions (Engaging with others, Collaboration and Open-mindedness) and nine sub-dimensions that potentially influence college students’ social and emotional competence are selected for this research. The description of the social–emotional competence is shown in Tables 3, 4. To assess the reliability and validity of the questionnaire used, the researchers conducted reliability and validity analyses on all the pre-test data of this research. The results indicated that the Cronbach’s alpha coefficients for each dimension ranged from 0.76 to 0.83, and the overall KMO coefficient was 0.76. This outcome demonstrated that the questionnaire used had good reliability and validity, as is shown in Table 4. The instrument employs a five-point Likert scale, with response options ranging from “strongly agree” (5 points) to “strongly disagree” (1 point), where higher scores indicate greater social and emotional competence. The survey is conducted in group sessions within the school’s office automation laboratory, ensuring standardized testing conditions.

**Table 3:** Description of the social–emotional competence domains.

Domains	Description
Engaging with others	Engaging with others is critical for leadership and tends to lead to better employment outcomes. People who score highly regarding “engaging with others” are energetic, positive and assertive. They also build social support networks more quickly, which is beneficial for mental health outcomes.
Collaboration	People who are open to collaboration can be sympathetic to others and express altruism. Collaboration translates into better quality relationships, more pro-social behaviors and less behavior issues.
Open-mindedness	Open-mindedness is also predictive of educational attainment, which has life-long positive benefits and seems to equip individuals better to deal with life changes.

**Table 4:** Description of the competence included in the survey on social–emotional competence.

Domains	Competence	Description	Cronbach’s α
Engaging with others	Energy	Approaches daily life with energy, excitement and spontaneity.	0.77
	Assertiveness	Able to confidently voice opinions, needs, and feelings, and exert social influence.	0.80
	Sociability	Able to approach others, both friends and strangers, initiating and maintaining social connections.	0.83
Collaboration	Empathy	Kindness and caring for others and their well-being that leads to valuing and investing in close relationships.	0.76
	Trust	Assuming that others generally have good intentions and forgiving those who have done wrong.	0.76
	Co-operation	Living in harmony with others and valuing interconnectedness among all people.	0.77
Open-mindedness	Tolerance	Is open to different points of view, values diversity, is appreciative of foreign people and cultures	0.76
	Curiosity	Interest in ideas and love of learning, understanding and intellectual exploration; an inquisitive mindset.	0.80
	Creativity	Generates novel ways to do or think about things through exploring, learning from failure, insight and vision	0.78

#### 3.2.3 Procedures

Researchers invited professional teachers from the school’s psychological center to conduct training for the organizers of the planned live action role-playing (LARP) games in March 2024. The training covered the DMs (dungeon master, which is originally appeared in the game Dungeon and Fighter, and later came to be generally referred to as the game master) and the NPC (non-player characters), who were primarily sourced from the school’s psychological health center staff and student volunteers from psychological health center clubs. The student volunteers participating in the organization of the activities volunteered to sign up, and were screened by psychology teachers. These students demonstrated strong willingness, outstanding language expression abilities, charisma, and appeal. During the training, psychology center teachers, along with the staff and students planning the activities, jointly conducted script readings, familiarizing themselves with the storylines, discussing relevant plot twists, and generating related written materials. Additionally, in accordance with the script requirements, they purchased relevant performance props and arranged the activity venues to match the scenes.

All subjects involved in the study were randomly assigned to two groups, an experimental group and a control group. The experimental group participated in an 8-week live action role-playing games, while the control group did not take part in any activities during this period. The organizers conducted the live action role-playing games for the experimental group from late March to May 2024. Unlike educational live action role-playing games conducted in classrooms for high school students, university students need to focus on professional learning in the classroom. Therefore, the project was carried out during the students’ convenient time, as a form of co-curricular activity. Except for statutory holidays, the activities were conducted once a week, with groups formed according to the number of participants required for the live action role-playing games. In addition to the participants, DMs and NPCs also took part in each activity. The duration of each session was primarily determined by the length of the script, but it also varied based on the improvisation of student role-playing during the event, generally lasting between 2 and 5 h. The organizers aimed to immerse university students emotionally through role-playing and incorporated unique interactive elements into some of the live-action role-playing games, such as reasoning, revealing mysteries, auctions, private conversations, and battles. These elements added entertainment and enjoyment, helping to prevent students from feeling bored during the role-playing activities. In China, popular LARPs typically feature one or more scenes focused on reasoning and identifying the murderer. However, the educational LARPs designed for this experiment incorporates additional elements beyond just reasoning. Some live action role-playing games had specific winning conditions; for example, in “Chongqing Mist,” each character had their own specific winning conditions, while others focused primarily on scene immersion, such as “Besieged,” where participants needed to make choices in a dilemma according to the plot. Both the experimental and control groups were assessed using measurement tools before and after the activities, and individual interviews were conducted after the experiment, each lasting 20–30 min. The scripts used in this study were selected by the research team based on the research objectives; they were well-regarded in the market and contained healthy content suitable for university student participation. Each live action role-playing game had different themes, as detailed in Table 5.

**Table 5:** Live action role-playing game scripts.

Week	Script name	Script type	Players	Script introduction
Week 1	Besieged	Emotion; War	7	The game is set in Shuoxian, Shanxi, China in 1937. Players take on roles such as the cavalry regiment commander, high school principal, president of a chamber of commerce, and other characters. The common goal of the characters is to defend the county town in the midst of war.
Week 2	An Isolated City	Bloc-to-bloc Confrontation	7	The game is set in a remote border town outside the Yumen Pass in China in 1931, which was a central gathering place for various forces during the period of the Republic of China. Players take on roles such as security guards, scholars, merchants, team leaders, and archeologists. The goal for players is to “survival of the fittest,” as they strive for survival in the struggle between the Light Camp and the Dark Camp, and to fulfill their own character objectives.
Week 3	Incoming Call	Emotion; Entertainment	6	The game is set in modern times, where players take on roles such as high school students, workers, teachers, and so on. Players earn initial capital by making money in various mini-games, and then complete a series of tasks through competition or cooperation.
Week 4	Scabbard	Bloc-to-bloc Confrontation	7	The game is set in Tianjin, China in 1948, where players take on roles such as the head of the secret service, doctors, and other characters, struggling to protect the country’s secrets.
Week 5	Mavlore Ship	Bloc-to-bloc Confrontation; War	6	The story takes place during the “Twilight of Calamity” when a giant ship named the Mavlore enters the ocean. Players take on roles such as the captain, priest, warrior, and others, forming alliances, fighting to seize the final treasure, and experiencing patriotism, family love, and romance in role-playing.
Week 6	Your Gift	Emotion; Urban Life	7	The story takes place in modern times, where players need to participate in an online live stream wedding of an elderly couple. Players take on roles such as the couple’s son, daughter, and others, working together to complete various fun game tasks, ultimately reconstructing the entire story.
Week 7	Northern Spring	Logic; Urban Life	7	The story takes place in a small border town in northern Ukraine in the 1980s, where players take on the roles of hardworking villagers. However, the tranquil atmosphere of the town is suddenly disrupted by an unexpected incident. Players need to collect evidence and deduce the true culprit, reconstructing the sequence of events that led to the incident.
Week 8	Every Day is a Good Day	Emotion; Ancient Customs	6	The story takes place in Tokyo, Japan, where players need to take on the roles of a married couple or siblings to participate in the recording of the program “Every Day is a Good Day,” and complete corresponding interactive tasks.

Quantitative data were collected from students in both groups before and after the experiment (i. e., after 8 weeks).

#### 3.2.4 Data analysis

Statistical analyses were conducted using SPSS version 26, with continuous variables presented as means (M) ± standard deviations (SD). The demographic data of the participants were analyzed. A Kolmogorov–Smirnov test for normality was used. The pre-test and post-test scores (means and standard deviations) of secondary indicators across three dimensions related to social–emotional competence were examined. A repeated-measures ANOVA test (time and group as factors) was performed to analyze the influence of the teaching methodologies on the dependent variables. The effect size (ES) was calculated by *η_p_^2^*, interpreted based on the following: small, moderate and large effect for values greater than 0.010, 0.059 and 0.138, respectively (Cohen, 2013). The alpha level was set at *p* < 0.05.

### 3.3 Qualitative research

#### 3.3.1 Participants

For the qualitative component, 14 participants (one-third of the total sample of the experimental group, *N* = 14) were selected through random sampling.

#### 3.3.2 Data collection

Qualitative data were collected by the principal researcher through individual semi-structured interviews and audio-recorded. The researcher designed qualitative interview questions separately for before and after the experiment, with some questions overlapping with the quantitative tests. This interview mainly focuses on two key aspects: “interest in games” and “self-evaluation of performance in each social–emotional competence dimension.” A total of 11 questions were set, as shown in Annex 2. The formulation of the interview questions strictly follows the principles of being clear, concise, and unambiguous, avoiding the use of overly professional or obscure words to ensure that interviewees can accurately understand the meaning of the questions. The design of the interview questions has referred to relevant research (Bowman and Standiford, 2015).

#### 3.3.3 Data analysis

For the processing of qualitative data, first of all, the researchers examined the responses to all the interview questions, classified the responses to each question into “yes,” “sometimes” and “no.” Secondly, thematic analysis was employed to explore the in-depth experiences and perceptions of participants. This method is highly suitable for uncovering latent themes within the rich qualitative data collected from the semi-structured interviews (Nowell et al., 2017) by using Nvivo software, we employed thematic analysis, a widely-used method in qualitative research. First, the audio-recorded interviews were transcribed verbatim. Then, the transcripts were repeatedly read to gain a familiarization with the data. Initial codes were generated by highlighting and labeling segments of text that related to the research questions, especially those related to students’ experiences, perceptions, and changes in social–emotional competence dimensions. For example, statements about how students interacted with others in the game, their feelings about teamwork, and their reactions to different opinions were coded.

After generating the initial codes, we grouped them into potential themes. These themes were refined and defined to ensure they accurately represented the data. For instance, themes related to the improvement of sociability in the “Engaging with others” dimension were identified, such as “opportunities for interaction with peers,” “learning from others in the game,” and “building confidence in communication.” In the “Collaboration” dimension, themes like “understanding others’ perspectives in role-playing,” “learning cooperation skills through tasks,” and “experiences of trust and betrayal” emerged. Regarding “Open-mindedness,” themes such as “tolerance for different viewpoints in the game,” “curiosity about game scenarios and characters,” and “perceived impact on creativity” were developed. The reliability and validity of the thematic analysis were ensured through several steps. Two researchers independently coded a subset of the data to check for consistency in coding. Any discrepancies were discussed and resolved through consensus-building. Also, during the theme development process, we constantly referred back to the original data to ensure that the themes were firmly grounded in the interviewees’ statements. This approach aligns with the guidelines for conducting rigorous thematic analysis (Nowell et al., 2017).

## 4 Results

### 4.1 Quantitative findings

Repeated measures analysis of variance was employed to examine the effects of group and time on the three primary dimensions (Table 6). For the dimension “Engaging with others,” the statistical results showed significant time-related differences with *F* = 13.813 (*p* < 0.001, *η_p_^2^* = 0.144). No significant group differences were observed (*F* = 1.106, *p* = 0.296, *η_p_^2^* = 0.013), but a significant group-time interaction emerged (*F* = 10.339, *p* = 0.002, *η_p_^2^* = 0.112). Simple effect analysis indicated no significant group main effect in the pre-test (*p* = 0.880) but a significant effect in the post-test (*p* = 0.031). The experimental group showed significant pre-post improvements (*p* < 0.001), while the control group did not (*p* = 0.724). For “Collaboration,” significant time effects were found (*F* = 5.716, *p* = 0.019, *η_p_^2^* = 0.065), with no group differences (*F* = 0.601, *p* = 0.440, *η_p_^2^* = 0.007), but a significant group-time interaction (*F* = 5.738, *p* = 0.019, *η_p_^2^* = 0.065). Simple effects revealed non-significant group main effects in both pre- and post-tests (*p* = 0.780, 0.109), but significant experimental group improvements (*p* = 0.001) and non-significant control group changes (*p* = 0.997). For “Open-mindedness,” significant time effects were observed (*F* = 4.024, *p* = 0.048, *η_p_^2^* = 0.047), with no group differences (*F* = 1.745, *p* = 0.190, *η_p_^2^* = 0.021), but a significant group-time interaction (*F* = 4.681, *p* = 0.033, *η_p_^2^* = 0.054). Simple effects showed non-significant group main effect in the pre-test (*p* = 0.821) and significant effect in the post-test (*p* = 0.015). The experimental group demonstrated significant pre-post improvements (*p* = 0.004), while the control group did not (*p* = 0.912). Overall, the non-significant group effects in the pre-test confirmed the homogeneity of experimental and control groups. After 8 weeks of LARP, the experimental group showed significant improvements in all three dimensions (Engaging with others, Collaboration, Open-mindedness) with statistically significant differences and moderate-to-large effect sizes (*η_p_^2^*), indicating enhanced social–emotional competence. The most pronounced improvement occurred in “Engaging with others” (*p* < 0.001).

**Table 6:** Results of repeated measures analysis of variance for the three primary dimensions.

Main dimension	Sub-dimension	Pre-test (M ± SD)	Post-test (M ± SD)		Time	Group	Time × Group
Engaging with others	Experimental Group	3.56 ± 0.40	3.72 ± 0.31 a^**^	*F*	13.813	1.106	10.339
	Control Group	3.54 ± 0.52	3.55 ± 0.39	*p*	<0.001	0.296	0.002
				*η_p_^2^*	0.144	0.013	0.112
Collaboration	Experimental Group	3.88 ± 0.43	4.01 ± 0.32^*^	*F*	5.716	0.601	5.738
	Control Group	3.90 ± 0.37	3.90 ± 0.33	*p*	0.019	0.440	0.019
				*η_p_^2^*	0.065	0.007	0.065
Open-mindedness	Experimental Group	3.82 ± 0.40	4.01 ± 0.32 a^*^	*F*	4.024	1.745	4.681
	Control Group	3.83 ± 0.38	3.82 ± 0.31	*p*	0.048	0.190	0.033
				*η_p_^2^*	0.047	0.021	0.054

^*^ or ^**^ represent significant differences between the post-test and pre-test of the experimental group in the simple effect analysis, where ^*^ indicates *p* < 0.05 and ^**^ indicates *p* < 0.001. “a” represents that there are significant differences between the data of the experimental group and the control group at the post-test time point in the simple effect analysis. The annotation of “a” means the p-value is less than 0.05.

This study further analyzed the secondary indicators of Engaging with others, Collaboration, and Open-mindedness in the experimental group’s pre-test and post-test. Repeated measures analysis of variance was used to analyze the effects of group and time on the nine secondary dimensions. Details can be found in Table 7. It could be seen from the *η_p_^2^* value and the *p* value that the scores for the six sub-dimensions—Assertiveness, Sociability, Empathy, Co-operation, Tolerance, and Curiosity—showed significant improvement, with statistically significant differences between pre-test and post-scores. Among them, the differences in the pre-test and post-test scores of the experimental group for the four secondary indicators of Assertiveness, Sociability, Empathy, and Co-operation were more significant (*p* < 0.001), and the improvements were more obvious.

**Table 7:** Results of repeated measures analysis of variance for the nine secondary dimensions.

Main dimension	Sub-dimension	Pre-test (M ± SD)	Post-test (M ± SD)	Statistical measure	Time	Group	Time × Group
Assertiveness	Experimental Group	3.48 ± 0.44	3.75 ± 0.35 a^**^	*F*	9.988	3.835	15.308
	Control Group	3.46 ± 0.50	3.43 ± 0.45	*p*	0.002	0.054	<0.001
				*η_p_^2^*	0.109	0.045	0.157
Sociability	Experimental Group	3.78 ± 0.52	4.01 ± 0.47 a^**^	*F*	13.819	1.582	11.736
	Control Group	3.75 ± 0.57	3.76 ± 0.49	*p*	<0.001	0.212	<0.001
				*η_p_^2^*	0.144	0.019	0.125
Energy	Experimental Group	3.40 ± 0.68	3.40 ± 0.54	*F*	0.339	0.071	0.530
	Control Group	3.41 ± 0.73	3.46 ± 0.61	*p*	0.562	0.790	0.469
				*η_p_^2^*	0.004	0.001	0.006
Empathy	Experimental Group	3.91 ± 0.57	4.13 ± 0.37 a^**^	*F*	4.265	0.770	7.704
	Control Group	3.96 ± 0.46	3.93 ± 0.51	*p*	0.042	0.383	0.007
				*η_p_^2^*	0.049	0.009	0.086
Trust	Experimental Group	3.81 ± 0.60	3.72 ± 0.50	*F*	1.0700	0.41600	0.3590
	Control Group	3.84 ± 0.49	3.82 ± 0.44	*p*	0.3040	0.52100	0.5510
				*η_p_^2^*	0.013	0.005	0.004
Co-operation	Experimental Group	3.90 ± 0.40	4.19 ± 0.44 a^**^	*F*	12.910	2.187	5.804
	Control Group	3.90 ± 0.43	3.96 ± 0.38	*p*	<0.001	0.143	0.018
				*η_p_^2^*	0.136	0.026	0.066
Tolerance	Experimental Group	4.00 ± 0.46	4.13 ± 0.40 a^*^	*F*	0.945	1.918	3.961
	Control Group	3.96 ± 0.46	3.91 ± 0.47	*p*	0.334	0.170	0.049
				*η_p_^2^*	0.011	0.023	0.046
Curiosity	Experimental Group	3.77 ± 0.60	4.01 ± 0.46 a^*^	*F*	2.053	1.125	4.165
	Control Group	3.81 ± 0.53	3.77 ± 0.59	*p*	0.156	0.292	0.044
				*η_p_^2^*	0.025	0.014	0.048
Creativity	Experimental Group	3.68 ± 0.49	3.89 ± 0.63	*F*	2.518	0.169	0.603
	Control Group	3.72 ± 0.57	3.79 ± 0.49	*p*	0.116	0.682	0.440
				*η_p_^2^*	0.030	0.002	0.007

^**^ or ^**^ represent significant differences between the post-test and pre-test of the experimental group in the simple effect analysis, where ^*^ indicates *p* < 0.05 and ^**^ indicates *p* < 0.001. “a” represents that there are significant differences between the data of the experimental group and the control group at the post-test time point in the simple effect analysis. The annotation of “a” means the *p*-value is less than 0.05.

### 4.2 Qualitative findings

In this experiment, 14 students were randomly selected from 42 students. The results revealed a large shift in social emotional ability as detailed in Table 8. “Yes” indicates that the social emotional ability is significantly improved, “Sometimes” indicates that students’ perception of improvement in their social–emotional abilities was inconsistent. The results of statistical description are presented in tabular form in the results section. In some game-playing scenarios or interactions, they felt that their abilities in the corresponding dimensions showed signs of enhancement, such as being more confident in expressing opinions or better at collaborating with others. However, in other situations, they did not notice any improvement or even felt that their performance regressed to some extent. “No” indicates no improvement or reduction.

**Table 8:** Descriptive statistics of semi-structured interview results.

Attitude tendency	Engaging with others			Collaboration			Open-mindedness		
	Assertiveness	Sociability	Energy	Empathy	Co-operation	Trust	Tolerance	Curiosity	Creativity
Yes	5 (35.71%)	7 (50.00%)	2 (14.29%)	2 (14.29%)	4 (28.58%)	4 (28.57%)	6 (42.86%)	7 (50.00%)	2 (14.29%)
Sometimes	6 (42.86%)	6 (42.86%)	2 (14.29%)	7 (50.00%)	5 (35.71%)	1 (7.14%)	7 (50.00%)	5 (35.71%)	4 (28.57%)
No	3 (21.43%)	1 (7.14%)	10 (71.42%)	5 (35.71%)	5 (35.71%)	9 (64.29%)	1 (7.14%)	2 (14.29%)	8 (57.14%)

#### 4.2.1 Engaging with others

After coding the data related to “Engaging with others,” several themes emerged, which is shown in Table 9. One prominent theme was “Variable Assertiveness Experiences.” This theme captured the diverse experiences of students regarding assertiveness. As seen from the interviews, some students, like Participant 7, faced setbacks when expressing opinions, with statements like “I have tried to express my opinion in a discussion and was ignored, and as a result, we did not complete the task together. It was not a successful experience for me, and I felt very frustrated.” This type of response was coded as instances of “assertiveness setbacks.” On the other hand, students such as Participant 6 had positive experiences, saying, “I bravely expressed my opinion during a discussion and led everyone to reason out the culprit. It was a successful experience. The games have given me the opportunity to speak up and express myself.” These were coded as “assertiveness achievements.” The co-existence of these two types of experiences within the data led to the identification of this theme, which reflects the heterogeneity in students’ assertiveness development during the live-action role-playing games.

**Table 9:** Thematic analysis results of “engaging with others.”

Theme category	Theme description	Typical quotations	Relevant sub-dimensions
Variable assertiveness experiences	Represents the diverse experiences of students when expressing opinions, including both positive and negative experiences.	Participant 7, Q4: “I have tried to express my opinion in a discussion and was ignored, and as a result, we did not complete the task together. It was not a successful experience for me, and I felt very frustrated.” Participant 1, Q4: “Once, I played the role of a leader, which means I need to persuade others to complete a task with me. I challenged myself, took the initiative to contact others, and several people agreed to join me, which brings me joy in leading others.”	Assertiveness
Enhanced sociability through interaction	Indicates that the games provided opportunities for students to interact with peers, helping them make friends, improve emotional intelligence and eloquence.	Participant 9, Q5: “Live action role-playing games have provided me with opportunities to interact with peers, enabling me to make friends and improve my emotional intelligence and eloquence.” Participant 6, Q5: “People have a wide range of styles when it comes to approaching tasks. I truly admire those outgoing individuals in games who have a knack for naturally uplifting the atmosphere. I intentionally observed and learned from them in the games, and in subsequent games, I also strived to become an active atmosphere builder myself.”	Sociability
Stable or decreased energy perception	Reflects that most students thought the games had no significant impact on their energy levels, and some even felt tired after playing.	Participant 5, Q3: “I do not think playing role-playing games has any significant impact on my vitality.” Participant 9, Q3: “Sometimes, a single game can last quite a long time, which can be somewhat tiring toward the end.”	Energy

Another theme was “Enhanced Sociability through Interaction.” Many interviewees described how the games provided opportunities for interaction. Participant 9 mentioned, “Live action role-playing games have provided me with opportunities to interact with peers, enabling me to make friends and improve my emotional intelligence and eloquence.” Statements like this, along with those about learning from others and building confidence in communication, were grouped under this theme. The games’ structured role-playing scenarios allowed students to engage with less-familiar individuals, which was a common aspect across multiple interviews. This theme highlights how the games contributed to students’ sociability by facilitating peer interaction and providing a platform for learning social skills.

Regarding the sub-dimension of Energy, the theme “Stable or Decreased Energy Perception” emerged. The majority of interviewees, such as Participant 5 who said, “I do not think playing role-playing games has any significant impact on my vitality,” and Participant 9 who mentioned, “Sometimes, a single game can last quite a long time, which can be somewhat tiring toward the end,” indicated that there was no clear improvement in energy. Some even felt tired after playing. These responses were coded accordingly, and the theme reflects the overall perception among students that the games did not enhance their energy levels, and in some cases, led to fatigue.

#### 4.2.2 Collaboration

When analyzing the data related to “Collaboration,” several themes emerged that provided insights into how the educational live action role-playing games affected students’ skills in this dimension, which is displayed in Table 10.

**Table 10:** Thematic analysis results of “collaboration.”

Theme category	Theme description	Typical quotations	Relevant sub-dimensions
Empathy development through role-playing	Demonstrates that students understood different characters and emotions during role-playing, including empathy from script-reading and real-time interaction.	Participant 9, Q6: “In the initial role allocation process, we are usually assigned to the roles that are more suitable for us to play. Therefore, we often find that roles can reflect ourselves. During the role-playing, I make an effort to understand the character and comprehend their behavioral style, which has led to a lot of experience in understanding people.” Participant 8, Q6: “I remember a host who was very professional, guiding us like an older brother and being very passionate at times, which deeply evoked intense emotions. During the game, many of us shed tears.”	Empathy
Enhanced cooperation awareness and skills	Shows that students recognized the importance of cooperation in the games and learned practical cooperation skills.	Participant 4, Q7: “Playing live action role-playing games requires a spirit of commitment. You cannot give up halfway, and everyone must follow the rules and make their own contribution; each person’s role is important.” Participant 11, Q7: “During the process of deducing the murderer, it’s essential for everyone to share their perspectives; the absence of any individual’s input can impact the final conclusion. Therefore, I often take the initiative to invite quieter participants to share their thoughts, as their contributions frequently prove to be pivotal.”	Co-operation
Heterogeneous Trust Experiences	Represents the diverse impacts of the games on students’ trust levels, including positive and negative experiences.	Participant 9, Q8: “In some missions, the murderer is one of the players. Instead of trusting teammates, I tend to believe in facts and evidence. At times, I feel like they have deceived me quite deeply.” Participant 6, Q8: “Subsequently, the facts proved that this person was indeed trustworthy, which allowed me to have a good gaming experience and gain confidence in interacting with others, making me more willing to trust others”	Trust

The first theme was “Empathy Development through Role-Playing.” This theme was derived from the interviews where students described their experiences of understanding different characters and emotions. As Participant 9 stated, “In the initial role allocation process, we are usually assigned to the roles that are more suitable for us to play. Therefore, we often find that the roles can reflect ourselves. During the role-playing, I make an effort to understand the character and comprehend their behavioral style, which has led to a lot of experience in understanding people.” This kind of response, along with others about empathizing with characters while reading the script, was coded as “script-based empathy.” In the real-time interaction aspect, Participant 5’s experience, “Once, during a game, a player made a surprising decision. Since then, I have come to understand that everyone has their own emotions and perspectives,” was coded as “interaction-based empathy.” These codes were grouped to form the theme, highlighting how the games facilitated the development of empathy among students.

Another theme was “Enhanced Cooperation Awareness and Skills.” Many respondents emphasized the importance of cooperation in the games. Participant 4’s statement, “Playing live action role-playing games requires a spirit of commitment. You cannot give up halfway, and everyone must follow the rules and make their own contribution; each person’s role is important,” was coded as “awareness of cooperation importance.” Participant 1’s experience of learning to be more patient in a cooperative task, “In a cooperative task, I was responsible for organizing all the clues but this experience inspired me to give others more time in collaborative tasks,” was coded as “learning cooperation skills.” These codes together indicated that students not only recognized the significance of cooperation but also acquired practical skills during the game.

Regarding the sub-dimension of Trust, the theme “Heterogeneous Trust Experiences” emerged. Some students, like Participant 7, “Some players in the game have presented a positive image which allowed me to have a good gaming experience and gain confidence in interacting with others,” had positive experiences that enhanced their trust. These were coded as “positive trust experiences.” On the other hand, Participant 9’s statement, “In some missions, the murderer is one of the players. Instead of trusting teammates, I tend to believe in facts and evidence,” and Participant 4’s experience of betrayal, were coded as “negative trust experiences.” The co-existence of these two types of experiences in the data led to the identification of this theme, showing the diverse impacts of the games on students’ trust levels.

#### 4.2.3 Open-mindedness

When analyzing the data related to “Open-mindedness,” distinct themes emerged for each sub-dimension, shedding light on how the educational live action role-playing games influenced students’ open-mindedness.

For the sub-dimension of Tolerance, the theme “Fostering Tolerance through Diverse Interactions” emerged. Participant 11’s experience, “Once, a player made a very passionate and lengthy argument, but the evidence wasn’t sound. However, I found that, like me, most players were able to patiently wait for him to finish speaking,” was coded as “tolerance in the face of unsound arguments.” Participant 7’s statement about changing their approach to handle different opinions, “When others voiced different opinions. I turned to wait for them to finish before sharing my own views,” was coded as “adapting communication for a tolerant atmosphere.” These codes, along with similar responses, formed this theme. It shows that through the games, students learned to understand and tolerate diverse voices, and actively sought ways to create a harmonious discussion environment.

Regarding Curiosity, the theme “Curiosity-Driven Exploration of Diverse Worlds” was identified. Participant 2’s enthusiasm for mystery scripts, “I particularly enjoy mystery scripts because I find the authors’ imagination to be so exquisite. I’m always curious about the answers to the mysteries in the scripts,” was coded as “curiosity about script content.” Participant 5’s desire to play different roles, “Originally, when choosing scripts. I also want to play characters that are somewhat different from myself to gain new perspectives,” was coded as “curiosity about different life experiences.” These codes were grouped to form this theme, indicating that the games’ diverse scenarios and roles inspired students’ curiosity, driving them to explore different storylines and gain new experiences.

For Creativity, the theme “Mixed Views on Creativity Enhancement” emerged. Participant 6’s admiration for the script’s creativity, “In some scripts I’ve encountered, the criminals’ modus operandi are so unique that they stretch my imagination,” was coded as “positive perception of creativity enhancement.” On the other hand, Participant 10’s view, “I feel that it does not really enhance creativity. The individuals who enhance creativity the most are likely the script developers,” was coded as “doubt about creativity enhancement.” The co-existence of these contrasting views in the data led to the identification of this theme, reflecting the varying impacts of the games on students’ creativity (Table 11).

**Table 11:** Thematic analysis results of “open-mindedness.”

Theme category	Theme description	Typical quotations	Relevant sub-dimensions
Fostering tolerance through diverse interactions	Indicates that students learned to understand and tolerate diverse voices through the games and created a harmonious discussion environment.	Participant 11, Q9: “Once, a player made a very passionate and lengthy argument, but the evidence wasn’t sound. However, I found that, like me, most players were able to patiently wait for him to finish speaking.” Participant 7, Q9: “When others voiced different opinions, I initially engaged in direct debates with them at first. However, I later realized that this would interrupt their speech, so I turned to wait for them to finish before sharing my own views, which helped create a more friendly atmosphere.”	Tolerance
Curiosity-driven exploration of diverse worlds	Reflects that the games’ diverse scenarios and roles inspired students’ curiosity, driving them to explore different storylines and gain new experiences.	Participant 2, Q10: “I particularly enjoy mystery scripts because I find the authors’ imagination to be so exquisite. I’m always curious about the answers to the mysteries in the scripts, and even after being told the truth behind the scenes, I still find them lingering in my mind.” Participant 9, Q10: “For instance, in emotional scripts, when a player expresses his affection for me, I am especially curious to know what events led to his devotion, and it fills me with curiosity.”	Curiosity
Mixed views on creativity enhancement	Represents the varying impacts of the games on students’ creativity, with some students believing it enhanced creativity while others doubted it.	Participant 6, Q11: “In some scripts I’ve encountered, the criminals’ modus operandi are so unique that they stretch my imagination, and deepen my admiration for the author’s creativity.” Participant 3, Q11: “Some endings have multiple unexpected twists, which to a certain extent have expanded my horizons and enhanced my creativity.”	Creativity

According to the qualitative research framework, from the proportion of “Yes,” “Sometimes” and “No” and the results of thematic analysis, it can be inferred that LARPs can improve the six sub-dimensions of Assertiveness, Sociability, Empathy, Co-operation, Tolerance and Curiosity in the social–emotional competencies of college students. As for Assertiveness, Trust and Creativity, participants’ feelings are heterogeneous, which provides researchers with richer information.

The investigators also analyzed the participants’ attitudes toward the LARPs. The vast majority of students held a positive attitude toward educational live action role-playing games. When asked about the most appealing aspect of these games, many students highlighted the immersive experience brought about by well-crafted scripts. They emphasized that a good script could draw them into the game world, allowing them to fully engage with their roles and enjoy the process of exploration and interaction. Second, some students expressed their affection for the game mechanics and innovative themes. Students enjoyed reasoning, decrypting, and working together with other players, as well as some wild script themes. Moreover, some students reported that the playing process made them feel more comfortable. Compared with other real social interactions, LARPs make them put down the pressure of daily social interaction. In the process of role-playing, they could express themselves more naturally, without worrying about too much self-exposure, and without feeling a strong didactic taste in the game.

However, when it came to the needed improvement of educational LARPs, students provided various suggestions. Some students hoped that the DMs (dungeon masters) could be more professional. They believed that professional DMs could better guide the game flow, handle unexpected situations, and help players have a more in-depth understanding of the game’s plot and rules. Others pointed out that the game scenarios could be made more elaborate and realistic. They thought that more vivid and detailed scenarios would enhance the overall atmosphere of the game and further immerse players. Additionally, a few students mentioned that the character settings in the games could be more diverse and balanced, so that every player could have a more fulfilling experience regardless of the role they played. These insights from students provide valuable directions for the future improvement of educational live action role-playing games. It should be noted that the thematic coding for this part of the content was rather scattered. Due to the diversity of themes and the complexity of the coding system, the specific coding is not presented here. This complexity reflects the rich and varied nature of students’ experiences and expectations regarding educational live action role-playing games, which provides valuable information for further research and improvement of such games.

### 4.3 Mixed research results

The mixed research results demonstrate parts of consistency derived from two experimental methods, as well as existing differences, and mention some supplementary content, as shown in Table 12.

**Table 12:** Combined display of the quantitative and qualitative findings.

Domains	Quantitative findings	Qualitative findings
Engaging with others	Post-tests of the experimental group showed an improvement in participants’ abilities, with improvements in the sub-skills of assertiveness and sociability.	In the dimension of assertiveness, substantial heterogeneity was observed. Some participants had experienced failures or had not attempted change, while others had received positive feedback and reported successful experiences after making changes. In the dimension of sociability, participants believed that educational live action role-playing games could provide an opportunity to interact with unfamiliar participants, easing the pressure of forming friendships. Additionally, they could observe and learn from others in educational live action role-playing games, gaining insights into everyday interactions. In terms of energy, the majority of respondents indicated that they did not experience any improvement in this aspect.
Collaboration	Post-tests of the experimental group showed an improvement in participants’ abilities, with improvements in the sub-skills of empathy and co-operation.	In the dimension of empathy, players sought to understand the characters and their emotions while reading the script, expressed their own feelings during activities, and were influenced by the emotions of others. In co-operation, players consciously followed game rules, recognized the importance of teamwork, and learned collaboration skills. In the dimension of trust, while some respondents reported an enhanced sense of trust, others recounted unpleasant experiences of betrayal.
Open-mindedness	Post-tests of the experimental group showed an improvement in participants’ abilities, with improvements in the sub-skills of tolerance and curiosity.	In the dimension of tolerance, players were able to understand and embrace the different positions of everyone in the game, tolerate different voices, and attempt to act in a friendly manner. In the dimension of curiosity, players explored different reasoning and experienced lives vastly different from their own, which led to positive experiences. Many also expressed a willingness to try more roles. The dimension of Creativity exhibited some variability among players.

The study adopted an explanatory sequential design, where the qualitative data is intended to explain and expand upon the quantitative findings. In this regard, the qualitative results offer valuable insights into the quantitative outcomes for each dimension. For the “Engaging with others” dimension, the quantitative improvement in assertiveness and sociability is further illuminated by the qualitative data. The theme of “Variable Assertiveness Experiences” reveals the diverse nature of students’ assertiveness development. The experiences of facing setbacks or achieving success when expressing opinions, as described in the qualitative analysis, explain why there is heterogeneity in the assertiveness improvement among students, which is consistent with the quantitative results. Regarding sociability, the theme “Enhanced Sociability through Interaction” shows how the games facilitate peer interaction. The opportunities for making friends and learning from others, as reported by the interviewees, provide a practical explanation for the observed increase in sociability skills in the quantitative assessment.

In the “Collaboration” dimension, the qualitative themes of “Empathy Development through Role-Playing” and “Enhanced Cooperation Awareness and Skills” align well with the quantitative improvements in empathy and co-operation. Through role-playing, students understand characters’ emotions and develop empathy, which is reflected in the increased scores of the empathy sub-dimension. Meanwhile, the emphasis on cooperation in the games and the acquisition of practical skills explain the improvement in co-operation. The “Heterogeneous Trust Experiences” theme clarifies the inconsistent results in the trust sub-dimension. The coexistence of positive and negative trust experiences in the game environment accounts for the lack of a clear overall improvement in trust, as seen in the quantitative data.

Regarding “Open-mindedness,” the qualitative themes “Fostering Tolerance through Diverse Interactions” and “Curiosity-Driven Exploration of Diverse Worlds” are consistent with the quantitative improvements in tolerance and curiosity. The ability to tolerate different viewpoints and the enthusiasm for exploring diverse scenarios in the games directly contribute to the growth in these two sub-dimensions. The “Mixed Views on Creativity Enhancement” theme, on the other hand, explains the variability in the creativity dimension among players in the quantitative results, indicating that the impact of the games on creativity is not uniform.

In summary, the integration of qualitative and quantitative data in this study, following the explanatory sequential design, provides a more comprehensive understanding of the impact of educational live action role-playing games on college students’ social–emotional competence. The qualitative findings not only validate the trends shown in the quantitative analysis but also offer in-depth explanations for the observed changes.

## 5 Discussions and implications

### 5.1 Discussions

This study, based on a pre- and post-test experimental comparison, found that live action role-playing games can enhance college students’ social–emotional competence in the dimensions of Engaging with Others, Collaboration, and Open-mindedness. Specifically, it improves six sub-dimensions: Assertiveness, Sociability, Empathy, Co-operation, Tolerance, and Curiosity. The results of the quantitative analysis are largely consistent with those from the one-on-one interviews.

The enhancement exhibited by students in the sub-dimension of Sociability corresponds to the discoveries documented in literature (Daniau, 2016; Fein, 2015; Henning et al., 2024). Likewise, the progress in the sub-dimension of Empathy (Hammer et al., 2018; Henning et al., 2024; Rivers et al., 2016) and co-operation (Adams, 2013; Guenthner and Moore, 2005; Guli et al., 2013) tallies with the findings presented in literature. According to the theory of dramatic simulation (Goffman, 2023), role-playing and scenario construction establish a performance framework which is distinct from reality. This framework efficaciously dismantles the power hierarchies present in real-life social interactions, such as those between educators and students or the senior and junior generations. It thereby affords students the opportunity to cultivate their social and emotional skills within an egalitarian environment. Face-to-face role-playing serves to obscure the demarcation between the front and back stages of the performance, endowing students with the capacity to concurrently embody the triple identities of performer, observer, and creator within the same spatio-temporal context. The emotional resonance mechanism, via the embodied experience of role immersion, spurs students to transcend their egocentric outlooks and gratify their emotional expression requirements. In terms of Co-operation, the scenario-based task design requires participants to set coordinated goals and complete tasks together. However, the competitive nature inherent in dramatic simulations (e.g., goal conflicts between roles) results in implicit resource competition among students, whereas trust establishment necessitates long-term interest alignment (Axelrod and Hamilton, 1981). This mechanistic contradiction explains the absence of significant improvement in the Trust sub-dimension.

In the sub-dimension of Assertiveness within “Engaging with Others,” the results of the quantitative research show a certain degree of improvement, which is consistent with the findings of references (Henning et al., 2024; Katō, 2019). However, some participants in the interviews reported a lack of subjective perception. This contradictory phenomenon requires in-depth analysis. Firstly, it can be explained from the differences in evaluation. In the quantitative research, part of the improvement in Assertiveness is reflected in explicit behavioral metrics (e.g., linguistic initiative frequency, conflict resolution dominance ratio, etc.). The lack of subjective perception reflected in the interview feedback may stem from the disconnection between self-awareness and behavioral performance. Secondly, the structural components of the game mechanics may determine the performance in terms of Assertiveness to a certain extent. According to the Thomas-Kilmann Conflict Mode Instrument, the design of resolving conflicts in resource allocation can trigger Assertiveness behaviors, whereas the absence of such game designs may lead to limited effects (Thomas, 2008). In this study, the experimental paradigm involved multiple components such as deductive reasoning, role-playing, and collaborative problem-solving dynamics. However, the competition mechanism may not be sufficient, which may explain this phenomenon. The way of role assignment also exerts an influence. According to Self-Determination Theory, if a role is endowed with higher decision-making authority or a more intricate growth trajectory, the need for autonomy can be fulfilled, its intrinsic motivation can be stimulated, the willingness for proactive behavior can be enhanced, and the sense of competence can be improved (Ryan and Deci, 2000). According to Self-Determination Theory, higher decision-making authority or a more intricate growth trajectory can fulfill the role’s demand for autonomy, trigger its intrinsic motivation, augment the inclination toward proactive behaviors and enhance the sense of competence. In Chinese context and in this experiment, the role assignment is predominantly executed by DM. During the role allocation process, the DM not only factors in the players’ personal preferences but also proactively assigns roles based on the players’ personalities, expressive capabilities, and game objectives. For illustration, a player possessing clear logical thinking is typically designated to assume the role of the “detective,” whereas an extroverted player is assigned to play the “villain” with the aim of intensifying the level of confrontation. This practice might prompt some players to stick to play the role of the “supporter,” while experienced players frequently take on the leadership role to steer the pace of the game. Such a phenomenon also unveils potential areas for enhancement in the design of subsequent educational LARPs. Looking ahead, the method of random drawing for role assignment could be contemplated, which enables participants to transcend their comfort zones and explore new possibilities. Finally, it can be analyzed from the perspective of cultural cognitive bias. In line with Triandis’ “Cultural Cognitive Bias Theory” (Triandis, 2018), the values of collectivism and modesty prevalent in Asian cultures potentially prompt participants to have a propensity to ascribe behavioral alterations to situational elements (Cai et al., 2020).

In the domain of Open-mindedness, the Tolerance sub-dimension within the experimental group demonstrated a significant improvement, corroborating the findings in references (Henning et al., 2024; Howes and Cruz, 2009; Pitkänen, 2015). The LARPs in this study furnished a relatively high degree of freedom, empowering players to depict and freely construe character images based on their personal insights. Moreover, the prevalent decision-making mechanisms in LARPs enabled participants to adeptly manage value conflicts within tight time constraints, nudging them toward compromise. Additionally, the fragmented narrative pattern of the game (Loewenstein, 1994) induced continuous information gaps and ignited the participants’ desire to solve the mystery. This spurred them to actively consolidate discrete information and vet multiple hypotheses, which plausibly accounts for the enhancement witnessed in the Curiosity sub-dimension.

The discoveries of this research have revealed the potential of educational LARPs within the Chinese higher education context. Through qualitative interviews, several salient advantages of LARPs can be encapsulated. Initially, immersive role-playing and team-based reasoning interactions constitute the most captivating elements of LARPs. This finding strongly resonates with prior research, signifying that these games endow participants with an “embodied experience,” cultivate “interest groups” in the sociological realm, present augmented social prospects, the interactivity of these games remedies the defect of unidirectional knowledge dissemination, which is a characteristic feature of traditional classrooms (Varela et al., 2017). Secondly, the structured narrative scaffold of LARPs functions as a “safe experimental arena” for individuals afflicted with social anxiety. Participants can subtly enhance their interpersonal abilities by assuming the roles of their in-game characters. They can experience a greater sense of relaxation and ease, unfettered by concerns of excessive self-disclosure, which is consistent with Abbott’s research (Abbott et al., 2022). Finally, the copious themes and variegated game modalities of LARPs markedly amplify college students’ incentive to engage. In contrast to traditional undertakings, LARPs aptly accommodate the personalized requisites and experience-emphasized traits of post-00s college students (Hongyu and Hu, 2022).

Nevertheless, the interviews have revealed the risks and limitations of educational LARPs. Firstly, it is indicated that a competent DM is of crucial importance for the effectiveness of LARPs. In Abbott’s research, two facilitators were set up, serving, respectively, as the DM and an ordinary player who could also supervise the interactions (Abbott et al., 2022). In this experiment, however, the staff only played the roles of the DM and Non-Player Characters (NPCs). Therefore, teachers need professional training to possess both the ability to guide and control the process, such as observing students’ performances in real time, controlling the rhythm, providing feedback, and creating a positive atmosphere. In subsequent research, it could be explored whether the staff should play the role of ordinary players to enhance the educational effect. Secondly, it is evident that significant differences exist in students’ participation. When students encounter unappealing themes or lack the willingness to act out the roles, their participation level may be low, which might potentially affect the educational outcome (Lave and Wenger, 1991). Thirdly, the time and resource costs are relatively high. When time conflicts arise due to team members’ other arrangements, the discussion and decision-making processes of some aspects may be hindered, and the level of immersion in role-playing will also be affected. Compared with group counseling which can be carried out by simply following a standardized process, scripted games have higher requirements for students’ level of engagement. In conclusion, careful design is needed in terms of script selection and DM guidance in order to meet the educational purposes.

The selection of well-crafted scripts with appropriate difficulty levels as pedagogical tools, combined with optimizing live-action role-playing (LARP) implementation methods to enhance college students’ social–emotional competencies, holds significant research value. However, standardized criteria for objectively evaluating script difficulty remain underdeveloped. Script difficulty perception constitutes a multidimensional construct influenced by interactive factors, such as narrative setting (e.g., fantasy/historical/sci-fi contexts), character complexity (personality/skill systems/interpersonal relationship networks), plot structure (narrative branching/suspense density), task typology (e.g., puzzle-solving/emotional engagement), and ending triggering mechanism (single ending or multiple endings/difficulty of achieving the conditions, etc.) Consequently, script difficulty assessment primarily relies on instructors’ pedagogical expertise and subjective evaluations, coupled with players’ personal gaming experiences and perceptions. Therefore, when selecting scripts, the organizers should place great emphasis on the quality and guard against the scripts being excessively entertaining or overly intricate, as these situations may cause a deviation from the pedagogical objectives and undermine the cultivation of social–emotional competencies (Hongyu and Hu, 2022). For example, researchers used the “Kids on Bikes” game and “Dungeons and Dragons” respectively as educational tools (Abbott et al., 2022; Varrette et al., 2023). In this study, eight scripts with different themes were selected and the quality was controlled. However, in the localization practice in China, how to select suitable scripts or create appropriate ones as educational tools remains an issue. On the one hand, educators, psychology experts, and scriptwriters are encouraged to engage in collaborative efforts to develop original scripts tailored to local college students’ cultural contexts, cognitive capacities, and social–emotional development objectives. Throughout this creative process, clearly defining specific social–emotional competency targets and aligning script narratives and character tasks accordingly. On the other hand, tools can be utilized to restructure and integrate existing scripts, improving the script quality. This ensures that while maintaining their fun and attractiveness, the scripts always closely revolve around the core goal of social–emotional skills cultivation, providing more targeted and effective tool support for the social–emotional education of Chinese college students (Lu, 2020).

Integrating live action role-playing games into educational environments is crucial for achieving sustainable development goals. The deep integration of education with the youth subculture of live action role-playing games brings significant educational value and contemporary relevance. It offers new insights for innovative and sustainable education in the modern era. Due to their immersive, interactive, and social nature, live action role-playing games enhance social–emotional competence and improve the effectiveness of education. These games encourage students to use their imagination and solve problems creatively. This approach is beneficial for developing adaptability, and comprehensive growth in knowledge, skills, and emotions. Incorporating sustainable education into live action role-playing games—such as designing scenarios around environmental or social issues—can further educate students and deepen their understanding of sustainable development principles. This aligns with the goals of sustainability.

### 5.2 Practical implications

The findings of this study offer significant implications for educational practice and curriculum design. Firstly, Based on the previous findings, the integration of educational LARPs into co-curricular programs emerges as a promising strategy to foster social–emotional competence among university students. From an international comparative perspective, South Korea’s “happiness education” and “free-semester system” have provided students with more optional courses and opportunities for social experiences, which also focuses on alleviating students’ examination pressure and cultivating their social adaptability. In comparison, the educational tool proposed in this paper has adopted the experiential learning concept of South Korea’s free-semester system, enabling students to practice and enhance their social and emotional competencies in an immersive and interactive environment. Current pedagogical approaches in Chinese universities predominantly rely on extracurricular activities (e.g., student associations/volunteer services) and group counseling to foster social–emotional competencies, yet both modalities exhibit significant limitations. While extracurricular initiatives lack systematic curation and professional mentorship (Jixu and Derun, 2023), group counseling struggles with participant disengagement stemming from uninspired activity designs and narrow thematic scopes (Yufang, 2022). This research provides evidence-based strategies to enhance learning outcomes through LARPs, proposing three critical enhancements: providing high-quality scenario scripts with pedagogical significance, creating immersive contextually authentic environments, and implementing professional development programs for facilitators. This evidence-based method addresses gaps by structuring experiential learning opportunities that align with students’ developmental needs while overcoming existing implementation challenges.

Secondly, this study offers frameworks for integrating gamified mechanisms and collaborative frameworks into curriculum design. Drawing on successful SEL projects from the US (Conley, 2015) and Singapore (Chong and Lee, 2015), educational stakeholders could adopt three strategies: (1) developing interdisciplinary courses that embed competitive-cooperative dynamics, (2) establishing tripartite partnerships among families, schools, and communities to reinforce learning outcomes, and (3) applying game design principles to create pedagogical materials that simultaneously advance cognitive and social–emotional development. For instance, integrating role-playing scenarios, team-based challenges, and reflective discussions into coursework could help students develop critical competencies such as empathy and communication. Such approaches align with the broader educational goal of preparing students for the complexities of the modern workforce, where social–emotional skills are increasingly valued.

Furthermore, the study underscores the value of combining quantitative and qualitative methods to assess educational effects. This mixed-methods approach not only validated the quantitative findings but also offered nuanced insights into students’ experiences and perceptions. Therefore, it is advisable for educators and researchers to adopt similar methodological frameworks when assessing the efficacy of innovative pedagogical strategies which ensure that both measurable outcomes and student voices are considered in the design and implementation of educational programs.

### 5.3 Limitations and future work

This research has several limitations that can be addressed in future studies.

Firstly, the representativeness of the sample was limited. Merely 84 college students were chosen, a relatively small size that might not mirror the diversity of Chinese college students. Hence, the generalizability of the conclusions needs further validation. Also, the study overlooked the potential influence of academic majors. In subsequent research, it is recommended to expand the sample size to include students from diverse regions, college types, and a broader range of academic majors, thereby enhancing the universality of the research findings. Furthermore, a comparative analysis could be conducted by categorizing students based on their academic majors. This would explore the unique pathways and effectiveness differences in enhancing different dimensions of social–emotional competence among students from disciplines such as liberal arts and engineering, which offers tailored recommendations for teaching practices.

Secondly, the experimental period was relatively short, and the frequency settings lacked flexibility. The 8-week experiment could only initially observe changes in social–emotional competence; it might be inadequate for cultivating deeper competencies and assessing long-term sustainability. For example, the research in reference (Thomas, 2008) indicates that the subjects were able to transfer the skills learned during the treatment process to the real world. However, in the quantitative experiment of reference (Sandstrom et al., 2022), the subjects in the intervention group lacked social confidence in their daily real-life, which suggests that overcoming social phobia is a relatively slow and gradual process. In future studies, it is recommended to extend the experimental period to one semester or even an entire academic year. Students’ social–emotional competence could be assessed at multiple stages, such as monthly and at the end of each semester, to track the developmental trajectory of their competencies and evaluate the long-term stability of the game’s effects. In terms of frequency settings, multiple experimental groups with varying frequencies of game participation could be established. This would enable a comparative analysis of the rate and persistence of competency improvement under different frequencies, thereby identifying the optimal frequency of game participation for educational practice. Furthermore, delayed tracking assessments of students’ social–emotional competencies should be implemented to examine the sustained effects of LARPs on students’ development. Additionally, the transferability of these competencies can be evaluated through behavioral indicators in subsequent interdisciplinary coursework, including structured observations of group collaboration dynamics and documented conflict resolution processes in mandatory courses.

Thirdly, the consideration of game design factors was not comprehensive enough. Although the current research has preliminarily revealed the enhancement of students’ social–emotional competence by LARPs, there are still significant limitations in the systematic analysis of game design elements. The existing conclusions mainly rely on the inter-group differences in pre- and post-test abilities and the personal perceptions of college students themselves. However, they overlook the multi-modal mechanism of LARPs as complex educational systems and fail to deeply explore the differential impacts of core design elements such as game content themes, difficulty levels, and interaction mechanism types on the development of social–emotional competence. In terms of future research, a game difficulty evaluation model that takes into account elements such as storyline complexity and ending triggering mechanisms could be considered to explore the differences in the impacts of different LARP difficulty levels on the social–emotional competence of college students. Besides, a factorial experimental approach could be systematically employed to analyze multiple game design factors. This would enable the construction of a social–emotional competence improvement model based on these elements, thereby providing scientific guidance for educational gamification practices.

## 6 Conclusion

This study collected performance data from Chinese college students before and after their participation in educational LARPs and employed a mixed-method approach to evaluate the effectiveness of LARPs in enhancing students’ social–emotional competence. The results revealed significant improvements in three key dimensions: Engaging with Others, Collaboration, and Open-mindedness, which highlights the educational potential of educational LARPs. Further analysis identified notable progress in six sub-dimensions: Assertiveness, Sociability, Empathy, Co-operation, Tolerance, and Curiosity. These findings suggest that integrating educational LARPs into college co-curricular activities offers a practical and effective approach to fostering social–emotional development. The immersive nature of these games encourages deep engagement, which enables students to refine their social–emotional competencies through experiential learning. Additionally, this study provides valuable insights for incorporating game-based elements and collaborative mechanisms into formal curricula, which could offer a foundation for designing classroom activities that incorporate role-playing and other interactive elements to enhance student learning outcomes.

## Data Availability

The original contributions presented in the study are included in the article/Supplementary material, further inquiries can be directed to the corresponding author.
