# Supplementary material for: The impact of educational live action role-playing games on social–emotional competence: a mixed-method study with Chinese college students
Source: Front Psychol. 2025 Jun 9;16:1538761. doi: 10.3389/fpsyg.2025.1538761 (PMC12188938; doi:10.3389/fpsyg.2025.1538761)
Supplement: Supplementary file 1 [file Supplementary_file_1.docx]

**Annex1：Questionaire**

**Part 1: Demographic Information**

1. Your Gender?
2. Your Age?
3. Your Region?
4. Your Major?
5. Your Grade?

**Part 2:** **Social and Emotional Competence Performance（The Likert five-point scale ：1 indicates strongly disagree, and 5 indicates strongly agree)**

6) I hardly ever feel a lack of energy.

7) I am capable of studying or working for extended periods.

8) My level of enthusiasm is high.

9) I can maintain a good level of energy throughout the day.

10) I enjoy taking on leadership roles in class and clubs.

11) When appointed as a group leader, I can easily and quickly accept and take on this responsibility.

12) I usually wait for others to take on leadership roles in a team.

13) When confronted with differing opinions, I prefer to stay silent.

14) I am confident in expressing my views in a team.

15) I know how to persuade others to follow my approach.

16) I enjoy spending time with my friends.

17) I am confident in establishing cooperation with strangers.

18) I communicate with my friends.

19) I avoid public speaking.

20) I feel stress when working in a large team.

21) When a classmate confides in me about their troubles, I feel impatient.

22) It is important to me that my friends are doing well.

23) I can understand the difficulties faced by classmates who work part-time while studying.

24) When I see someone feeling down or crying, I feel distressed.

25) I am confident in understanding what others desire.

26) I believe that most people are honest.

27) I believe that most people have good intentions.

28) When interacting with friends, I feel that I cannot trust them well.

29) When my friends make mistakes, I can forgive them.

30) When encountering difficulties in academic studies or club activities, I am likely to actively seek help from classmates, seniors, or teachers.

31) I am good at listening to the opinions of my classmates.

32) I feel skilled at getting along with other people.

33) When my classmates and I have differing opinions, I am able to reach a consensus through negotiation.

34) I advocate mutual knowledge sharing and collaborative learning among classmates.

35) When the group requires me to make compromises, I find it easy to accept.

36) I enjoy learning about different cultures and religions.

37) I find it hard to get along with classmates who have different family backgrounds from mine.

38) I am able to accept the individual differences among classmates.

39) I find that I can understand viewpoints and values that differ from my own.

40) Regardless of my classmates' actual performance, I can identify the strengths in each of them.

41) I enjoy learning new things at school.

42) I like traveling to new destinations.

43) I don’t like unknown changes.

44) I am not fond of exploring the functions of new products.

45) I can propose solutions that others do not think of.

46) I have good imagination.

47) I am able to learn from failures, insights, and foresight.

48) I am confident in creating works of value.
