# Supplementary material for: The impact of educational live action role-playing games on social–emotional competence: a mixed-method study with Chinese college students
Source: Front Psychol. 2025 Jun 9;16:1538761. doi: 10.3389/fpsyg.2025.1538761 (PMC12188938; doi:10.3389/fpsyg.2025.1538761)
Supplement: Supplementary file 2 [file Supplementary_file_2.docx]

| **Serial Number** | **Summary of Interview Questions** |
| --- | --- |
| 1 | Do you like the form of LARP? |
| 2 | What is your favorite part of LARP games? What is the part you dislike the most? And how do you think it could be improved? |
| 3 | After playing the LARP game, do you feel more energetic? |
| 4 | Do you think you are a good leader in a team? Has the LARP game been helpful for you to play a leadership role? Did you take the initiative to speak up during the discussion session? How did you feel? |
| 5 | Do you get along well with other classmates? Has the LARP game helped you get along better or worse with other classmates? What inspiration does the LARP game bring to you in terms of getting along with classmates? |
| 6 | After playing the LARP game, do you think you've got a better understanding of others? |
| 7 | In your opinion, do you work well with other classmates in the LARP game? Has the LARP game contributed to enhancing your teamwork with other classmates in daily life? |
| 8 | Through playing the LARP game, are you more willing to trust others? |
| 9 | In the LARP game, when others express opinions different from yours, what kind of choice do you make? And how do you perceive those who hold different viewpoints from you? |
| 10 | In the LARP game, do you have a strong curiosity about other characters? Does this make you more willing to try new things or more resistant to trying new things? |
| 11 | Do you have the feeling that your creativity has been enhanced? |

**Annex Table.** Semi-structured interview questions
